# Clinical manifestations and outcomes of fetal periventricular pseudocysts: a study of 38 cases

**DOI:** 10.3389/fped.2026.1842530

**Published:** 2026-07-13

**Authors:** Jing Ding, YiHan Xiao, Jie Fu, Jia Liu, Yifang Yuan, Qiuyan Pei

**Affiliations:** 1 Department of Pediatrics, Peking University People’s Hospital, Beijing, China; 2 Department of Pediatrics, Shandong Provincial Hospital Affiliated to Shandong First Medical University, Jinan, Shandong, China; 3 Clinical Research Institute, Institute of Advanced Clinical Medicine, Peking University, Beijing, China; 4 Department of Gynecology and Obstetrics, Peking University People’s Hospital, Beijing, China

**Keywords:** clinical manifestations, fetal periventricular pseudocysts, outcomes, pregnancy, ultrasound

## Abstract

**Objective:**

This study aimed to describe the clinical manifestations and enhance the understanding of outcomes linked to fetal periventricular pseudocysts.

**Methods:**

We conducted a retrospective evaluation of 38 pregnant women diagnosed with fetal periventricular pseudocysts between January 2015 and May 2025.

**Results:**

Among the participants, five women were diagnosed during the second trimester, while 33 were diagnosed in the third trimester. Of the total 38 women, three opted for therapeutic abortion due to congenital abnormalities, while 35 delivered (two preterm and 33 at term). The average gestational age at first diagnosis was 31.4 (29.3, 34.6) weeks. In particular, seven periventricular pseudocysts were left-sided, six were right-sided, and 25 were bilateral. Twenty-three of these pseudocysts were situated on the lateral aspect of the anterior horn of the lateral ventricle. Throughout the course of pregnancy, 15 of the 38 pseudocysts were closely monitored, demonstrating variable changes in size. Three fetuses were found to have genetic or chromosomal abnormalities during pregnancy. In addition, two newborns required admission to the neonatal intensive care unit due to infection and prematurity. Among the 34 newborns, two exhibited developmental delays in language and/or motor domains.

**Conclusions:**

Most fetal periventricular pseudocysts were detected in the second and third trimesters, with over two-thirds being bilateral. The lateral aspect of the anterior horn of the lateral ventricle was the primary site of occurrence. With the exception of cases accompanied by other abnormalities, the majority of fetal periventricular pseudocysts were associated with favorable outcomes.

## Introduction

Periventricular pseudocysts (PVPCs) are small, fluid-filled cavities located adjacent to the brain ventricles, characterized by the absence of the typical lining found in true cysts (1). These pseudocysts are encased in germinal matrix cells and glial components. PVPCs are believed to arise from antenatal cystic regression of the germinal matrix, or may be associated with bleeding, tissue infarction, or congenital viral infections (2). Advances in ultrasound (US) and magnetic resonance imaging (MRI) technology have led to a gradual rise in the detection rates of fetal PVPCs, with prevalence estimates ranging from 0.5% to 5.2% among infants assessed using cranial ultrasound (3, 4).

Upon receiving a diagnosis of fetal PVPCs, the primary concern for pregnant women frequently pertains to prognosis. Although several studies have investigated the outcomes associated with PVPCs, uncertainties concerning their clinical relevance persist. Esteban et al. investigated the prenatal features of isolated subependymal pseudocysts and proposed that a PVPC with a major axis exceeding 9 mm, particularly when located near the occipital and temporal horns, behind the caudothalamic notch, or exhibiting unusual morphology, may indicate poor outcomes (5). Conversely, Cooper et al. found no meaningful link between the morphological characteristics observed on magnetic resonance imaging and neurodevelopmental outcomes (6). Moreover, Sun et al. found that PVPCs often decrease in size or resolve completely after birth. Generally, isolated PVPCs are associated with normal postnatal outcomes, regardless of their location, number, or size. However, PVPCs that are accompanied by additional findings tend to be linked to poorer neurodevelopmental outcomes compared with isolated cases (1, 7).

The inconsistencies regarding the prognosis of PVPCs present challenges for antenatal consultations and may exacerbate anxiety among pregnant women. Therefore, it is crucial to examine the timing of diagnosis, morphological characteristics, temporal changes, and neurodevelopmental outcomes associated with PVPCs. This study seeks to characterize the clinical manifestations and outcomes of fetal periventricular pseudocysts identified at our center over the past decade, thereby offering valuable insights into this condition.

## Materials and methods

### Patients

In this retrospective analysis, we examined electronic medical records of pregnant women diagnosed with fetal periventricular pseudocysts at Peking University People's Hospital between January 2015 and May 2025. All procedures adhered to the ethical guidelines outlined in the Helsinki Declaration of 1975, as amended in 2013.

### Fetal ultrasound examination procedure

Fetal ultrasound examinations were performed using the HERA W9 system (Samsung, South Korea) and the Voluson E10 system (GE Healthcare, USA), both equipped with transabdominal convex array transducers. Transducer frequencies were 3.5–5.0 and 1–8 MHz, respectively. Systematic fetal assessments were conducted in accordance with the International Society of Ultrasound in Obstetrics and Gynecology (ISUOG) guidelines for midtrimester fetal ultrasound screening (8).

For cranial evaluation, three standard imaging planes were obtained: the transventricular plane, the transthalamic plane, and the transcerebellar plane. The primary structures assessed included cerebral anatomy, lateral ventricles, choroid plexus, cavum septi pellucidi, midline falx cerebri, thalami, cerebellum, and cisterna magna. Examinations were conducted by sonographers with at least 5 years of clinical experience. Confirmatory diagnoses were established through joint review by two examiners.

Fetal periventricular pseudocysts were diagnosed via ultrasonography, which revealed anechoic or hypoechoic, rounded, non-communicating cystic lesions situated near the lateral ventricles, specifically in the frontal horn and adjacent temporal or occipital horns, without extending above the external angle of the lateral ventricle (9).

### Treatment

Some fetal periventricular pseudocysts were monitored dynamically, and none underwent additional interventions during pregnancy or after birth.

### Statistical analysis

Continuous variables were presented as mean ± standard deviation for data with a normal distribution, while non-normally distributed data were reported as medians along with interquartile ranges. Comparisons were conducted using independent samples *t*-tests or the Mann–Whitney U test depending on the context. Categorical variables were analyzed with the *χ*^2^ test or Fisher's exact test. Statistical analyses were performed using SPSS version 20.0 for Windows, with the significance level set at *p* < 0.05.

## Results

### Characteristics of the pregnant women diagnosed with fetal periventricular pseudocysts

Thirty-eight pregnant women were identified with fetal periventricular pseudocysts, comprising five cases identified during the second trimester and 33 during the third trimester. Within this cohort, five pregnancies underwent fetal MRI, which confirmed the diagnosis made by prenatal ultrasound. Among these 38 women, three opted for therapeutic abortion, while 35 delivered, including two preterm and 33 full-term newborns. The therapeutic abortions were performed due to fetal conditions, in particular, polycystic kidney disease, bone dysplasia, and cerebellar hypoplasia. The average gestational age upon diagnosis was 31.4 weeks (29.3 weeks, 34.6 weeks). The average age of the 38 expectant mothers was 31 years (28 years, 35 years). None of the mothers or neonates were infected with cytomegalovirus (CMV). Detailed information regarding these 38 cases of fetal periventricular pseudocysts is presented in Table 1.

**Table 1:** Characteristics of the pregnant women diagnosed with fetal periventricular pseudocysts.

Patient no.	Age	Gestational age at diagnosis	Complex anomalies	Treatment during pregnancy	Delivery or abortion	Full-term or preterm	Maternal infection	Neonatal infection	Genetic or chromosomal test
1	35–40	34.6	Polycystic kidney disease	None	Abortion	/	None	None	NIPT
2	40–45	33.1	None	None	Delivery	Full-term	None	None	SNP, Karyotype
3	30–35	34.3	IUGR, small pericardial effusion, persistence of the cavum vergae, dilation of the fourth ventricle, left lateral ventricular enlargement	None	Delivery	Full-term	None	None	SNP, Karyotype, WES
4	30–35	30.7	None	None	Delivery	Full-term	None	None	SNP, Karyotype
5	30–35	35	None	None	Delivery	Full-term	None	None	SNP, Karyotype
6	35–40	39.1	None	None	Delivery	Full-term	None	None	NIPT
7	20–25	35.3	None	None	Delivery	Full-term	None	None	NIPT
8	30–35	33.9	None	None	Delivery	Full-term	None	None	SNP, Karyotype
9	25–30	30.4	None	None	Delivery	Full-term	None	None	SNP, Karyotype
10	25–30	36.4	None	None	Delivery	Full-term	None	None	NIPT
11	20–25	31.1	None	None	Delivery	Full-term	None	None	None
12	25–30	27.3	None	None	Delivery	Full-term	None	None	SNP, Karyotype
13	25–30	30.3	None	None	Delivery	Full-term	None	None	NIPT
14	30–35	29.1	arr (1–22) × 2, (XN) × 1	None	Delivery	Full-term	None	None	SNP, Karyotype
15	30–35	29.1	None	None	Delivery	Full-term	None	None	None
16	20–25	36.3	None	None	Delivery	Full-term	None	None	None
17	30–35	31.6	None	None	Delivery	Full-term	None	None	SNP, Karyotype
18	25–30	32.7	None	None	Delivery	Full-term	None	None	None
19	25–30	22.7	None	None	Delivery	Full-term	None	None	SNP, Karyotype
20	30–35	31.4	None	None	Delivery	Full-term	None	None	SNP, Karyotype
21	35–40	32.1	Bone dysplasia	None	Abortion	/	None	None	SNP, Karyotype
22	30–35	29.4	None	None	Delivery	Full-term	None	None	SNP, Karyotype
23	30–35	31.7	None	None	Delivery	Full-term	None	None	SNP, Karyotype
24	20–25	31.4	None	None	Delivery	Full-term	None	None	NIPT
25	40–45	28.1	46,XN,inv(9)(p13q13)	None	Delivery	Full-term	HSV IgM+	None	SNP, Karyotype
26	30–35	31.1	None	None	Delivery	Full-term	None	None	None
27	30–35	27.4	None	None	Delivery	Full-term	None	None	SNP, Karyotype
28	30–35	36	None	None	Delivery	Full-term	None	None	NIPT
29	25–30	35.4	None	None	Delivery	Full-term	None	None	NIPT
30	35–40	29.3	None	None	Delivery	Full-term	None	None	SNP, Karyotype
31	30–35	30	None	None	Delivery	Full-term	None	None	SNP, Karyotype
32	35–40	28	FECH, c.315–48 T > C	None	Delivery	Preterm	None	None	WES
33	25–30	31	None	None	Delivery	Full-term	None	None	SNP, Karyotype
34	25–30	35.6	None	None	Delivery	Full-term	None	None	NIPT
35	30–35	35	None	None	Delivery	Full-term	HSV IgM+	None	NIPT
36	20–25	32	None	None	Delivery	Preterm	None	None	NIPT
37	30–35	27.9	None	None	Delivery	Full-term	None	None	SNP, Karyotype
38	40–45	25	Cerebellar hypoplasia	None	Abortion	/	None	None	NIPT, WES

IUGR, intrauterine growth restriction; HSV, herpes simplex virus; SNP, single nucleotide polymorphism array; WES, whole exome sequencing; NIPT, non-invasive prenatal testing.

### Clinical manifestations of the fetal periventricular pseudocysts

**Figure 1:**
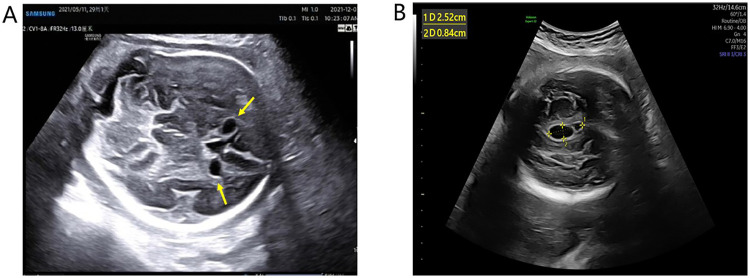
Periventricular pseudocysts. **(A)** Two unilocular periventricular pseudocysts were observed on the lateral aspect of the anterior horn of the lateral ventricle on both sides at 30^+2^ weeks of gestation. **(B)** At 35 weeks of gestation, bilocular periventricular pseudocysts measuring 2.52 × 0.84 cm were identified on the left side (yellow marker).

Among the 38 fetal periventricular pseudocysts identified, seven were located on the left side, six on the right side, and 25 were bilateral. Twenty-three of the cysts were located on the lateral aspect of the anterior horn of the lateral ventricle, while seven were situated in the subependymal region. Six pseudocysts were found on the anterior horn of the lateral ventricle, one was positioned anteroinferior to the lateral ventricle, and another was located superolateral to the anterior horn of the lateral ventricle. The sizes of the fetal periventricular pseudocysts at initial diagnosis ranged from 0.3cm × 0.4 cm to 2.6cm × 1.5 cm (Figure 1). Fifteen of the 38 cysts were monitored throughout the course of pregnancy, revealing variable changes in characteristics. One fetus exhibited persistence of the cavum vergae (sixth ventricle), dilation of the fourth ventricle, and left lateral ventricular enlargement. Detailed information regarding the characteristics of the fetal periventricular pseudocysts is presented in Table 2.

**Table 2:** Clinical manifestations of the fetal periventricular pseudocysts.

Patient no.	Side	Location	Numbers	Size changes
1	Right	Anterior horn of the lateral ventricle	3	/
2	Left	Lateral aspect of the anterior horn of the lateral ventricle	1	/
3	Bilateral	Anterior horn of the lateral ventricle	1 + 1	/
4	Bilateral	Lateral aspect of the anterior horn of the lateral ventricle	1 + 1	Increase first and then decrease on both sides
5	Bilateral	Lateral aspect of the anterior horn of the lateral ventricle	1 + 1	/
6	Bilateral	Lateral aspect of the anterior horn of the lateral ventricle	1 + 2	/
7	Bilateral	Lateral aspect of the anterior horn of the lateral ventricle	1 + 3	Decrease on both sides
8	Bilateral	Subependymal	3 + 4	Increase on both sides
9	Left	Lateral aspect of the anterior horn of the lateral ventricle	1	/
10	Bilateral	Anteroinferior to the lateral ventricle	1 + 1	/
11	Right	Lateral aspect of the anterior horn of the lateral ventricle	1	/
12	Bilateral	Lateral aspect of the anterior horn of the lateral ventricle	1 + 1	Decrease on both sides
13	Bilateral	Lateral aspect of the anterior horn of the lateral ventricle	1 + 1	Increase on both sides
14	Bilateral	Lateral aspect of the anterior horn of the lateral ventricle	1 + 1	Decrease first and then increase on both sides
15	Bilateral	Anterior horn of the lateral ventricle	1 + 1	Increase on both sides
16	Left	Subependymal	1	/
17	Bilateral	Lateral aspect of the anterior horn of the lateral ventricle	1 + 1	/
18	Left	Lateral aspect of the anterior horn of the lateral ventricle	1	Decrease
19	Left	Anterior horn of the lateral ventricle	1	/
20	Right	Subependymal	1	/
21	Bilateral	Lateral aspect of the anterior horn of the lateral ventricle	1 + 1	/
22	Right	Anterior horn of the lateral ventricle	1	Increase
23	Bilateral	Anterior horn of the lateral ventricle	3 + 1	/
24	Bilateral	Superolateral to the anterior horn of the lateral ventricle	2 + 1	/
25	Bilateral	Lateral aspect of the anterior horn of the lateral ventricle	1 + 1	Increase first and then decrease on both sides
26	Left	Lateral aspect of the anterior horn of the lateral ventricle	1	Increase
27	Bilateral	Lateral aspect of the anterior horn of the lateral ventricle	1 + 1	Decrease on both sides
28	Bilateral	Lateral aspect of the anterior horn of the lateral ventricle	1 + 1	/
29	Bilateral	Subependymal	1 + 1	/
30	Bilateral	Lateral aspect of the anterior horn of the lateral ventricle	1 + 1	/
31	Bilateral	Lateral aspect of the anterior horn of the lateral ventricle	1 + 1	The left side increases, the right side decreases
32	Right	Lateral aspect of the anterior horn of the lateral ventricle	1	/
33	Bilateral	Subependymal	1 + 1	/
34	Bilateral	Subependymal	1 + 1	/
35	Left	Lateral aspect of the anterior horn of the lateral ventricle	1	Increase first and the decrease
36	Bilateral	Lateral aspect of the anterior horn of the lateral ventricle	1 + 1	/
37	Right	Lateral aspect of the anterior horn of the lateral ventricle	1	Decrease
38	Bilateral	Subependymal	1 + 1	/

In the numerical column, “1 + 2” signifies the presence of one periventricular pseudocyst on the left side and two periventricular pseudocysts on the right side.

### Pregnancy outcomes

Of the 38 pregnant women diagnosed with fetal periventricular pseudocysts, three opted for therapeutic abortion, while 35 proceeded to delivery. Among these deliveries, 26 were vaginal and nine were cesarean section. Two pregnant women tested positive for herpes simplex virus IgM on TORCH infection screening; however, none of the infants demonstrated evidence of TORCH infection on subsequent evaluation. The average gestational age at the time of delivery was 38.6 (38, 39.4) weeks. Two women had preterm deliveries, while 33 delivered at term. Of the 38 fetuses, 33 underwent one or more genetic and chromosomal testing modalities, including non-invasive prenatal testing (NIPT), single nucleotide polymorphism (SNP) array analysis, karyotyping, or whole-exome sequencing (WES), with comprehensive details provided in Table 1. Three infants were identified as having genetic or chromosomal abnormalities during pregnancy. In particular, one infant had a karyotype of 46,XN,inv(9)(p13q13), another exhibited an SNP result of arr (1–22) × 2, (XN) × 1, and a third infant carried a variant in the porphyria-related gene FECH, c.315-48 T > C.

### Prognosis of infants with fetal periventricular pseudocysts

The average birth weights and lengths of the infants were 3,230 g (2,990, 3,455) and 50 cm (49, 50.5), respectively. All infants were delivered alive, with four classified as small for gestational age. One infant received a 1-min Apgar score of less than 8, while all 5-min Apgar scores exceeded 8. Two infants were transferred to the neonatal intensive care unit (NICU) due to infection and prematurity. Neurodevelopmental follow-up was conducted by telephone at approximately 24 months of age or corrected age. When standardized developmental assessment results were available, Bayley-Ⅲ or Ages and Stages Questionnaire (ASQ-3) results were recorded. Two infants were identified as having developmental delays involving language and/or motor domains based on available Bayley-Ⅲ results. For the remaining infants, no developmental concerns were reported by parents during telephone follow-up. The infant exhibiting both language and motor developmental delays (Bayley-Ⅲ, language composite score: 78, motor composite score: 80) had prenatal ultrasound findings that indicated intrauterine growth restriction (IUGR), small pericardial effusion, persistence of the cavum vergae, dilation of the fourth ventricle, and left lateral ventricular enlargement; however, genetic and chromosomal tests for this infant were normal. The infant with isolated language delay (Bayley-Ⅲ, language composite score: 82) showed no abnormalities other than periventricular pseudocysts, with both genetic and chromosomal evaluations returning normal results. Among the three infants with identified genetic or chromosomal abnormalities, neurological outcomes remained within the normal range. Detailed prognostic information for the 35 infants diagnosed with fetal periventricular pseudocysts is provided in Table 3.

**Table 3:** Prognosis of infants with fetal periventricular pseudocysts.

Infant no.	Birth GA (weeks)	Birth weight (g)	Birth length (cm)	1 min Apgar	5 min Apgar	NICU admission	Neurological prognosis
1	38	2,500	48	10	10	No	Normal
2	40^+^^2^	2,600	50	10	10	No	Speech and motor delay
3	39^+^^5^	3,710	52	9	10	No	Normal
4	40	4,000	51	10	10	No	Normal
5	37^+^^6^	2,820	49	10	10	No	Normal
6	38^+^^3^	3,460	50	10	10	No	Normal
7	38^+^^6^	3,370	50	10	10	No	Normal
8	39^+^^2^	3,510	51	8	10	No	Normal
9	37^+^^5^	2,980	50	10	10	No	Normal
10	38^+^^4^	2,850	48	10	10	No	Normal
11	38^+^^1^	3,640	50	10	10	No	Normal
12	38^+^^1^	3,260	51	10	10	Yes	Normal
13	38^+^^3^	3,220	49	10	10	No	Normal
14	39	3,450	52	10	10	No	Normal
15	40	3,550	50	10	10	No	Normal
16	39^+^^2^	3,230	50	10	10	No	Normal
17	39^+^^3^	3,380	50	9	10	No	Normal
18	40^+^^3^	3,350	50	10	10	No	Speech delay, motor normal
19	38^+^^5^	3,190	51	10	10	No	Normal
20	38^+^^2^	3,060	50	10	10	No	Normal
21	37^+^^5^	2,930	49	10	10	No	Normal
22	38^+^^2^	3,570	50	10	10	No	Normal
23	41	3,350	50	10	10	No	Normal
24	39^+^^5^	3,220	49	10	10	No	Normal
25	39^+^^2^	3,140	48	10	10	No	Normal
26	38^+^^2^	2,360	46	7	9	No	Normal
27	37^+^^3^	3,000	49	10	10	No	Normal
28	38^+^^4^	3,320	50	8	10	No	Normal
29	37^+^^6^	3,060	50	10	10	No	Normal
30	39^+^^1^	3,890	51	10	10	No	Normal
31	36	2,570	45	10	10	No	Normal
32	39^+^^3^	3,970	51	10	10	No	Normal
33	38	3,250	52	10	10	No	Normal
34	36	2,000	46	10	10	No	Normal
35	37^+^^6^	3,200	50	10	10	No	Normal

GA, gestational age; NICU, neonatal intensive care unit.

### Characteristics of isolated and non-isolated periventricular pseudocysts

As presented in Table 4, PVPCs were categorized into isolated and non-isolated groups, with 89.5% classified as isolated. Adverse outcomes, including therapeutic abortion and neurodevelopmental abnormalities, were observed predominantly among non-isolated PVPCs. The clinical characteristics of the two cases exhibiting neurodevelopmental abnormalities are presented in Supplementary Table S1.

**Table 4:** The characteristics of isolated and non-isolated PVPCs.

Characteristics	Isolated PVPCs (*n* = 34)	Non-isolated PVPCs (*n* = 4)
Mother's median age (year)	31	38
Median GA at diagnosis (week)	31.1	33.2
Abortion, *n* (%)	0	3 (75)
Full-term, *n* (%)	32 (94.1)	1 (25)
Neurodevelopmental abnormalities, *n* (%)	1 (2.9)	1 (25)

PVPC, periventricular pseudocysts; GA, gestational age.

## Discussion

This study involved a retrospective analysis of data from 38 pregnant women diagnosed with fetal periventricular pseudocysts during pregnancy. Our findings indicated that the majority of fetal periventricular pseudocysts were detected during the second and third trimesters, with more than two-thirds being bilateral. The lateral aspect of the anterior horn of the lateral ventricle was the primary site of occurrence. In this cohort, except for cases with additional abnormalities, most fetal periventricular pseudocysts appeared to demonstrate favorable outcomes.

Upon confirmation of a fetal periventricular pseudocyst diagnosis, pregnant women often expressed concerns about whether prior healthcare providers may have overlooked the condition. Our study revealed that the average gestational age upon diagnosis was 31.4 weeks, with the majority of cases occurring between 28 and 32 weeks of gestation, consistent with the existing literature (9). Periventricular pseudocysts arise in the germinal matrix during its swift progression at the onset of the second trimester and during the subsequent phase of rapid lysis toward its conclusion (5). This temporal association may explain their detection in the second and third trimesters. Another important consideration is the potential impact of this diagnosis on the risk of preterm birth. Our findings demonstrated that 94.3% of the fetuses were carried to full term. It appears that fetal periventricular pseudocysts may not be associated with an increased likelihood of prematurity, which affects approximately 12% of pregnancies globally (10). However, further studies are needed to validate this conclusion.

Color Doppler imaging of periventricular pseudocysts typically reveals no detectable flow, with the lesions appearing as well-circumscribed, anechoic, and non-vascular areas (11). In our cohort, 65.8% of the periventricular pseudocysts were bilateral. Notably, we observed similar incidence rates for unilateral left-sided and right-sided PVPCs. In addition, some of the periventricular pseudocysts exhibited a multilocular structure. Approximately 60.5% of the cysts were located on the lateral aspect of the anterior horn of the lateral ventricle, while other identified locations included the anterior horn of the lateral ventricle, subependymal regions, anteroinferior to the lateral ventricle, and superolateral to the anterior horn. With the progression of diagnostic technology, fetal MRI is being increasingly utilized for the evaluation of fetal intracranial disorders. In our cohort, five cases underwent fetal MRI, which corroborated the diagnosis of PVPCs. Yasar et al. analyzed 104 fetuses with postnatal verification and found that fetal MRI achieved significantly better diagnostic performance than ultrasonography (92.9% vs. 76.8%), with the greatest incremental benefit for central nervous system (CNS) abnormalities (12). Other studies similarly demonstrated that fetal MRI provides a substantial increase in diagnostic accuracy and additional diagnostic information over ultrasound for CNS anomalies (13–16). Accordingly, when prenatal screening identifies PVPCs or other CNS abnormalities, it may be reasonable to recommend a follow-up fetal MRI, provided it is feasible.

At initial diagnosis, the sizes of the fetal periventricular pseudocysts ranged from 0.3 cm × 0.4 cm to 2.6 cm × 1.5 cm. Among the 38 cysts, 15 were monitored throughout the course of pregnancy, revealing variable changes in their characteristics. In these cases, some periventricular pseudocysts decreased in size, while others increased, with some exhibiting fluctuations. The observed changes in size during pregnancy may be associated with the underlying pathophysiology of pseudocyst formation.

Prior investigations have linked periventricular pseudocysts with chromosomal microdeletions (such as 4p-) along with metabolic and mitochondrial pathologies, underscoring the importance of genetic consultation and diagnostic evaluations, encompassing karyotype assessment and chromosomal microarray analysis (17, 18). In our cohort, 7.9% of the cases were found to have genetic or chromosomal abnormalities. An additional 7.9% of the pregnant women opted for therapeutic abortion due to other complex congenital dysmorphisms. Most cases were characterized as isolated periventricular pseudocysts without accompanying dysmorphic features; however, adverse outcomes were observed primarily in non-isolated PVPCs.

Among the 35 deliveries, 74.3% were vaginal, indicating that fetal periventricular pseudocysts may not elevate the likelihood of cesarean delivery. Prognosis for fetal periventricular pseudocysts remains a primary concern, largely influenced by factors such as site, dimensions of the lesions, etiology, and the existence of related anomalies (1, 19). In our study, all newborns were delivered alive, with only one experiencing perinatal asphyxia, demonstrating that the presence of these pseudocysts may not correlate with increased mortality. Moreover, one infant necessitated admission to the NICU due to an infection unrelated to the pseudocysts. The average birth weights and lengths of the newborns fell within normal ranges, despite four infants being designated as small for gestational age, with some experiencing fetal growth restriction.

Concerning long-term neurodevelopment outcomes, we noted that two infants exhibited developmental delays; one presented with additional brain abnormalities, while the other did not. Overall, our findings indicate that there should not be excessive concern regarding the prognosis of periventricular pseudocysts in the lack of accompanying abnormalities. This observation may be accounted for by prior evidence indicating that isolated PVPCs are not independently linked to neonatal white matter microstructural alterations (2).

### Strengths and limitations

This study summarizes key clinical features of fetal periventricular pseudocysts identified at our center over the past decade and provides valuable insights into their prognosis. Nonetheless, this study presents several limitations. First, the relatively small sample size may have affected the reliability of our results to some degree. Second, not all fetuses underwent ultrasound dynamic monitoring during pregnancy. Third, in evaluating the neurological development of infants, we primarily relied on parental feedback via telephone interviews and the ASQ-3, neither of which constitutes a diagnostic test. Future work should include prospective studies and the use of the Bayley-Ⅲ scale to support further validation.

## Conclusions

In summary, most fetal periventricular pseudocysts were identified during the second and third trimesters, with over two-thirds exhibiting a bilateral presentation. The predominant location of the pseudocysts was the lateral aspect of the anterior horn of the lateral ventricle. Dynamic monitoring throughout pregnancy revealed variability in the sizes of the pseudocysts. A subset of infants demonstrated genetic or chromosomal abnormalities alongside other complex congenital dysmorphisms. In this cohort, most fetal periventricular pseudocysts appeared to have favorable outcomes on follow-up, except in cases with additional abnormalities.

## Data Availability

The raw data supporting the conclusions of this article will be made available by the authors, without undue reservation.
