# Supplementary material for: Clinical manifestations and outcomes of fetal periventricular pseudocysts: a study of 38 cases
Source: Front Pediatr. 2026 Jul 13;14:1842530. doi: 10.3389/fped.2026.1842530 (PMC13402555; doi:10.3389/fped.2026.1842530)
Supplement: Supplementary file 1 [file Supplementaryfile1.docx]

Table S1. Description of PVPCs with neurodevelopmental abnormalities

| Case | Gestational age at diagnosis | Gestational age at birth | Size | Laterality | Localization | Associated anomaly | Outcome |
| --- | --- | --- | --- | --- | --- | --- | --- |
| 1 | 34.3 | 40.3 | 1.2cm×0.6cm (right)  1.4cm×0.7cm (left) | bilateral | Anterior horn of the lateral ventricle | IUGR, small pericardial effusion, persistence of the cavum vergae, dilation of the fourth ventricle, left lateral ventricular enlargement | Speech and motor delay |
| 2 | 22.7 | 40.4 | 1.3cm×0.5cm | left | Anterior horn of the lateral ventricle | None | Speech delay, motor normal |
